# Planned Oocyte Cryopreservation Following the 2021 French Bioethics Law: Women’s Characteristics and Early Clinical Outcomes From a Single‐Center Observational Study

**DOI:** 10.1155/jp/7642675

**Published:** 2026-09-06

**Authors:** Camélia Taibi, Nathalie Rives, Maria Letailleur, Fanny Jumeau, Noémie Kauffmann, Romane Levade, Aurélie Feraille

**Affiliations:** ^1^ Biology of Reproduction-CECOS Laboratory, Rouen University Hospital, Rouen, France, univ-rouen.fr; ^2^ Univ Rouen Normandie, Inserm U1239, NorDIC, team “Adrenal and Gonadal Pathophysiology”, Biology of Reproduction-CECOS Laboratory, Rouen University Hospital, Rouen, France, univ-rouen.fr; ^3^ Centre Clinique d’Assistance médicale à la procréation, service de gynécologie-obstétrique, Rouen University Hospital, Rouen, France, univ-rouen.fr

**Keywords:** fertility preservation, oocyte cryopreservation, planned oocyte cryopreservation, social egg freezing, woman fertility

## Abstract

**Background and Aims:**

Planned oocyte cryopreservation (POC) has been authorized in France since 2021 within a specific national counselling and reimbursement framework. The primary aim of this study was to describe the sociodemographic characteristics, motivations, and fertility‐related knowledge of women seeking POC after implementation of the 2021 French Bioethics Law. Secondary aims were to report early cycle outcomes and explore factors associated with oocyte vitrification outcomes.

**Methods:**

We conducted a descriptive, observational single‐center study at Rouen University Hospital, retrospectively analyzing prospectively collected standardized counselling data and POC cycle outcomes. Women registering for POC between September 1, 2021, and December 31, 2022, were included, with follow‐up through July 31, 2023. Associations between available clinical and biological parameters and oocyte vitrification outcomes were explored.

**Results:**

Overall, 117 women registered for POC, and 69 attended the POC counselling consultation. Participants had higher educational levels than the general French population; the leading motivations were single status and awareness of age‐related decline in ovarian reserve. By July 31, 2023, 40 women had initiated COH treatment and 38 had completed at least one full cycle. The mean number of cryopreserved mature metaphase II oocytes was 12.1 ± 8.8 per woman. Exploratory analyses showed that AMH and AFC were positively associated with oocyte vitrification outcomes, whereas higher BMI was associated with lower oocyte yield and reduced maturation rate.

**Conclusion:**

In this early single‐center French cohort, women seeking POC were mostly highly educated and were mainly motivated by single status and awareness of age‐related fertility decline. Early vitrification outcomes were associated with ovarian reserve markers and BMI. Longer‐term follow‐up is required to assess oocyte utilization, pregnancy and live birth outcomes, and the impact of publicly funded POC on access to fertility preservation in France.

## 1. Introduction

In France, the average age at first childbirth was 28.9 years in 2020 [1], up from 24.2 years in 1967 [2]. This increase in the age at first childbirth is also observed across the European Union, where each country has observed a similar trend [2]. This delay in maternal age can be attributed to various factors, such as the legalization of contraception, the predominant presence of women in higher education, a discrepancy in perception between “social” and “reproductive” age linked to a lack of knowledge about ovarian reserve, and an excessive reliance on Assisted Reproductive Technology (ART) [1, 3]. The critical age of 31 years appears to mark a decline in the probability of conceiving per cycle, which decreases from 25% at age 25 to 12% at age 36, and further to 6% at age 42 [4]. Even with the aid of ART, age remains a negative predictive factor for pregnancy likelihood. According to data from the French Biomedicine Agency, the birth rate per oocyte retrieval decreased from 27.6% before age 30 to 14.4% at ages 38–39, and to 3.2% from the age of 43 [5]. Finally, the risk of spontaneous miscarriage becomes 2.4 times greater from the age of 40 years and the risk of chromosomal abnormalities in oocytes is 9.9 times higher from the age of 40 years [6].

The development of ART procedures including oocyte vitrification had led to a marked improvement in the efficacy of oocyte cryopreservation (OC) and had rendered possible fertility preservation in women exposed to gonadotoxic treatment [7]. However, OC has also been proposed even in women who were not facing a fertility‐threatening disease but as a potential procedure for those who have not yet completed their parental project and who want to postpone it. This procedure is generally referred to as “social egg freezing” or “oocyte freezing for non‐medical reasons” or “planned oocyte cryopreservation” (POC). The Ethics Committee of the American Society for Reproductive Medicine (ASRM) has introduced the term “planned oocyte cryopreservation” to describe fertility preservation method using “elective” egg freezing that is “undertaken as a matter of planning before a medical indication has materialized” [8]. In addition, the fourth version of the French Bioethics Law promulgated in August 2021 legalized “gamete self‐preservation for women and men […] without medical indication and without a prerequisite for previous donation” (French bioethics law n°2021‐1017, 2021), permitted POC from 29 to 37 years and ART use of cryopreserved oocytes up to 45 years [9, 10]. Financially, while controlled ovarian hyperstimulation (COH), ovarian puncture, OC, and cryopreserved oocyte use through ART are fully covered by the French health insurance, annual storage cost (€40 per year) is fully charged to beneficiaries. Additionally, private or public companies are prohibited from bearing the cost or providing compensation in any form for planned gamete cryopreservation by their employees [11]. The French financial support is completely different from other countries where such comprehensive financial coverage by health insurance is not typically available, making France unique in its support for POC.

Several studies concerning POC have been conducted across various countries, analyzing different aspects of POC from public opinions to live births obtained from cryopreserved oocytes, including COH cycle outcomes based on women age. Regarding opinions on POC, one survey conducted among 1049 Belgian women of reproductive age, using an email questionnaire, revealed that 31.5% of participants were considering POC [12]. In France, one study surveyed 547 women aged 18 to 74 regarding POC before the French Bioethics law was presented to the Parliament. Among women under 35 years, 19.3% expressed a desire to have access to POC, and for women over 35 years, this demand increased to 37.2%. Furthermore, approximately three‐quarters of the respondents indicated they would choose to freeze their oocytes between the ages of 25 and 30 years. Moreover, the primary motivation cited was “completing education and/or establishing a professional career and/or gaining independence from parents” (between 79.8% and 57.1%). The majority of women favored partial or income‐based reimbursement for the procedure [13]. In contrast, a recent study conducted in France found that the main motivation among women who had actually undergone POC was a desire for pregnancy while being single, reported by 60% of participants. Advancing one’s professional career ranked only fourth, cited by just 6% of the women [14].

In addition, some studies have used questionnaires on personal and relational status, knowledge about fertility, and the motivations of women who have undergone POC. These studies were mostly single‐center and conducted retrospectively after POC, exploring populations of predominantly heterosexual, educated, and unmarried women who regretted not being sufficiently informed beforehand about age‐related fertility decline by healthcare professionals [14–35]. The primary information sources for women who cryopreserved their eggs for social reasons are mainly the media (internet, television, magazines), friends, and, to a lesser extent, healthcare professionals [14, 15, 22, 27, 30, 33, 36]. Furthermore, most women express very little regret about their decision to undergo POC, with regret rates ranging from 0% to 16%, and 95% of patients would be willing to undergo the procedure again [18, 25, 31, 33, 37]. Finally, several women indicated they would be willing to donate their unused frozen oocytes for research purposes, with reports ranging from 22% to 63% [16, 22, 25, 30, 33, 35]. Additionally, in a less extent, some women would consider donating their cryopreserved oocytes for others women (from 4.8% to 31%) [16, 22, 25, 30, 33, 35].

The outcomes of POC reported in various international ART centers indicated that the average age at oocyte retrieval was around 37 years [16, 18–20, 22, 23, 25–36, 38–40]. Women typically underwent an average of 1 to 3 POC cycles, resulting in significantly variable numbers of cryopreserved oocytes per woman from one study to another [31, 34, 38, 39, 41, 42]. Specifically, the number of cryopreserved oocytes decreased with age: from 9.6–12.8 for ages 26–30 to 8.6–9.6 for ages 31–35, and finally to 5.6–6.6 for ages 36–40 [39, 41]. Despite this decline, the oocyte maturation rate remained stable at 80% across all age groups [41]. The reuse rate of cryopreserved oocytes varies significantly across different studies (3%–38%) and depends on the duration of follow‐up [16, 18, 20–23, 25, 31, 33–35, 40, 42–48]. However, post‐thaw survival and fertilization rates of frozen‐thawed oocytes did not seem to be influenced by the age at the time of OC [42].

Ultimately, the live birth rate was negatively correlated with the patient’s age at the time of POC (17%–31%) and with the number of cryopreserved oocytes [20, 33, 34, 38–40, 42–48].

In accordance with the data of these studies, Goldman et al. [49] developed a model intended as a counseling tool for centers involved in fertility preservation. According to this model, at the age of 35 years, it would be necessary to cryopreserve 20 mature oocytes to achieve a 90% chance of a live birth, while at the age of 37 years, more than 30 oocytes would be required [49].

POC activity, now authorized in France, has been already established in several countries that have published descriptive studies of this activity. The primary objective of our study was to describe the sociodemographic characteristics, motivations, and fertility‐related knowledge of women seeking POC after implementation of the 2021 French Bioethics Law in a publicly funded setting. The secondary objectives were to report early clinical outcomes of POC cycles and to explore associations between clinical and biological parameters and oocyte vitrification outcomes.

## 2. Materials and Methods

### 2.1. Study Design and Population

We conducted a single‐center observational retrospective study based on prospectively collected data to report on POC activity at the Reproductive Biology Laboratory‐CECOS of Rouen University Hospital. This study was reported in accordance with the STROBE (Strengthening the Reporting of Observational Studies in Epidemiology) guidelines for observational studies, and the completed STROBE checklist for cohort studies is provided as Supplementary File 1 (**STROBE_checklist_Taibi_JOP**). Eligible patients included all women who contacted the Reproductive Biology Laboratory‐CECOS to register on the waiting list for a POC counselling consultation between September 1, 2021, and December 31, 2022. Data collection began with the opening of registration on the waiting list, following the promulgation of bioethics law No. 2021‐1017 on August 2, 2021 [11]. Consultations started on January 24, 2022, after the publication of Decree No. 2021‐1933 of the Public Health Code on December 31, 2021, and data collection continued until July 31, 2023 [9, 10].

The POC counselling consultation was conducted by a reproductive medicine specialist. First, each woman completed a standardized questionnaire addressing lifestyle, motivations, knowledge about POC, and medical and gynecological history. All questions were multiple‐choice, with an option for free‐text responses to provide additional explanatory details (Table 1 and Table 2).

**Table 1:** Sociodemographic, reproductive, and professional characteristics of women attending the POC counselling consultation.

Personal situation	
**Q1 What is your age? mean ± SD (min-max) (years)**	

34.6 ± 1.9 (29.6–37)	
**Q2 What is your personal situation? n (%)**	

Married: 3 (4.3)	Cohabiting: 11 (15.9)
Divorced: 4 (5.8)	Engaged in a relationship without cohabitation: 11 (15.9)
Engaged in a civil partnership: 2 (2.9)	Single: 38 (55.1)

**Q3 What is your sexual orientation? n (%)**	
Heterosexual: 64 (92.8)	
Homosexual: 4 (5.8)	
Bisexual: 1 (1.4)	

**Q4 Where do you live? n (%)**	
Large city (> 100,000 inhabitants): 41 (59.4)	
Small town (< 100,000 inhabitants): 21 (30.4)	
Countryside: 7 (10.1)	

**Q5 Do you live alone? n (%)**	
Yes: 49 (71)	
No: 20 (29)	

**Q6 What is your accommodation? n (%)**	
House: 21 (30.4)	Owner: 44 (63.8)
Apartment: 48 (69.6)	Hosted: 1 (1.4)
Tenant: 24 (34.8)	

**Q7 How many children do you have? n (%)**	
0:63 (91.3)	
1:6 (8.7)	
> 1:0	

**Q8 Is your current partner the father of your children? n (%)**	
Yes: 1 (16.7)	
Previous partner: 5 (83.3)	

**Q9 Are they under your responsibility? n (%)**	
Yes: 6 (100)	

**Q10 Do you have a current desire for pregnancy? n (%)**	
Yes: 29 (42)	
No: 40 (58)	

**Q11 If you currently have no desire to become pregnant, how long do you think it will be before you plan to have a child? mean ± SD (range: min-max) (years)**	
2.95 ± 1.3 (1.5–5)	
Professional situation	

**Q12 What is your profession? n (%)**	
Medical: 20 (28.9)	
Other: 49 (71.1)	

**Q13 Have you completed higher education? n (%)**	
Yes: 66 (95.7)	
No: 3 (4.3)	

**Q14 If you have, what is your level of education? n (%)**	
High school: 3 (4.3)	Fourth‐year university level: 5 (7.2)
Second‐year university level: 7 (10.1)	Fifth‐year university level: 25 (36.2)
Third‐year university level: 14 (20.3)	More than a fifth‐year university level: 15 (21.7)

**Q15 What degree do you have? n (%)**	
Vocational certificate: 3 (4.3)	PhD: 8 (5.8)
Two‐year technical degree: 5 (7.2)	Engineer: 9 (13)
Bachelor’s degree: 15 (21.7)	Other: 9 (13)
Master’s degree: 25 (36.2)	

**Q16 Have you reached your professional goal? n (%)**	
Yes: 48 (69.5)	
No: 21 (30.5)	

**Q17 If not achieved, do you think it is important to achieve this goal before having a child? n (%)**	
Yes: 8 (38.1)	
No: 13 (61.9)	

Abbreviations: max: maximum; min: minimum; *n*: number; POC: planned oocyte cryopreservation; SD: standard deviation.

**Table 2:** Motivations, knowledge, perceptions, and future intentions regarding POC among women attending the counselling consultation.

Fertility preservation motivations	
**Q18 Who referred you to the Reproductive Biology Laboratory-CECOS? n (%)**	

Own initiative: 31 (44.9)	Gynecologist: 24 (34.8)
General practitioner: 3 (4.3)	Other: 6 (8.7)
Midwife: 5 (7.2)	

**Q19 How did you hear about social fertility preservation? n (%)**	
Internet/social networks: 26 (37.7)	Family and friends: 33 (47.8)
News (TV/Newspapers): 9 (13)	Doctor: 23 (33.3)

**Q20 Are there any fertility problems in your family? n (%)**	
Yes: 32 (46.7)	
No: 37 (53.6)	

**Q21 What circumstances influenced your decision to consider oocyte preservation? n (%)**	
Information about ovarian reserve: 48 (69.6)	Pressure of biological clock: 20 (29)
Celibacy: 33 (47.8)	Societal pressure: 5 (7.2)
Beginning of a relationship with no immediate plan for children: 19 (27.9)	Financial pressure: 3 (4.3)
Professional: 14 (20.3)	Infertility of family and friends: 2 (2.9)

**Q22 Have you told your friends and family about your project? n (%)**	
Yes: 68 (98.6)	
No: 1 (1.4)	

**Q23 How did they react? n (%)**	
Favorably: 60 (87)	
Negatively: 9 (13)	

**Q24 Does your employer encourage you to carry out this oocyte preservation? n (%)**	
Yes with financial compensation: 0 (0)	
Yes without financial compensation: 2 (2.9)	
No: 67 (97.1)	

**Knowledge on oocyte conservation**	

**Q25 In your opinion, what is the maximum age at which oocytes should be stored in order to have a chance of pregnancy? mean ± SD (range: min-max) (years)**	
37.7 ± 2.5 (35‐45)	

**Q26 In your opinion, what is the maximum age for using cryopreserved oocytes to have a chance of pregnancy? mean ± SD (range: min-max) (years)**	
42.9 ± 2.8 (37‐55)	

**Q27 Do you consider oocyte cryopreservation to be a 100% guarantee/assurance of having a child one day? n (%)**	
Yes: 5 (7.2)	
No: 64 (92.8)	

**Q28 Do you think that oocyte cryopreservation is a risky medical procedure? n (%)**	
Yes: 34 (49.3)	
No: 35 (50.7)	

**Q29a Do you think that medical care during fertility preservation is at your financial expense? n (%)**	
Yes: 12 (17.4)	
No: 57 (82.6)	

**Q29b Do you think that long-term gamete storage during fertility preservation is at your financial expense?n (%)**	
Yes: 55 (79.7)	
No: 12 (20.3)	

**Q30 Would you have carried out the same procedure if medical care for oocyte preservation was not reimbursed by your health insurance? n (%)**	
Yes: 49 (71)	
No: 20 (29)	

**Oocytes reuse**	

**Q31 If you need to reuse your gametes in the future, do you plan to do so: n (%)**	
In a relationship: 41 (59.4)	In a relationship/alone with sperm donation: 15 (21.7)
Alone with sperm donation: 6 (8.7)	Do not know: 7 (10.1)

**Q32 If you do not use your gametes for your own purposes, have you considered: n (%)**	
Donation to a couple: 18 (26.1)	Destruction: 11 (15.9)
Donation to research: 17 (24.6)	Do not know: 2 (2.8)
Donation without preference: 21 (30.4)	

Abbreviations: CECOS: Centre de conservation des œufs et du sperme; max: maximum; min: Minimum; *n*: number; POC: planned oocyte cryopreservation; SD: standard deviation.

Secondly, the physician explained the French regulations and the practical clinical and biological steps of the OC procedure, including the risks associated with COH treatment and oocyte retrieval. The woman was informed about the success rates of POC, including the number of cryopreserved oocytes required according to her age to achieve a 90% chance of a live birth, based on the model by Goldman et al. [49]. An assessment of ovarian reserve, preferably performed on Day 3 of the menstrual cycle, included measurement of basal serum levels of follicle‐stimulating hormone (FSH, IU/L), luteinizing hormone (LH, IU/L), 17*β*‐estradiol (E_2_, pg/mL), anti‐Müllerian hormone (AMH, ng/mL), and a pelvic ultrasound for antral follicle count (AFC). For women using hormonal contraception, only AMH measurement and AFC evaluation were performed. This assessment was combined with standard serological testing for human immunodeficiency virus (HIV), hepatitis B and C, and syphilis.

Finally, women who proceeded with the POC process attended a second consultation to confirm the feasibility of the procedure and to determine the appropriate COH protocol, including the type and starting dose of gonadotrophins, based on their medical history, ovarian reserve, age, and weight. All patients with complete medical records were reviewed during a weekly multidisciplinary team meeting involving reproductive medicine specialists and reproductive biologists, to validate the indication for POC and confirm the absence of medical contraindications to COH and oocyte retrieval.

A target number of oocytes to be cryopreserved per woman was proposed based on age, according to the model by Goldman et al. [49]: the goal was 20 oocytes for women under 35 years of age and 30 oocytes for those aged 35 and older.

### 2.2. COH, Follicular Puncture, and Oocyte Vitrification

The COH treatment could include recombinant FSH alpha (FSHr *α* No.1: GONAL‐F [Merck Europe B.V., Amsterdam, Netherlands]; FSHr *α* No.2: BEMFOLA [Gideon Richter, Budapest, Hungary]), recombinant FSH beta [PUREGON (N.V. Organon, Kloosterstraat 6, Netherlands)], or a combination of recombinant FSH alpha and recombinant LH alpha [PERGOVERIS (Merck Europe B.V., Amsterdam, Netherlands)]. Daily injections were administered by the patients themselves or with the assistance of a home nurse.

The first ultrasound examination and hormonal monitoring were performed on the sixth day of stimulation. The gonadotropin dose was adjusted based on the patient’s response. In the case of an antagonist protocol, a daily injection of GnRH antagonist [FYREMADEL 0.25 mg (Ferring Pharmaceuticals, Saint‐Prex, Switzerland); ORGALUTRAN 0.25 mg (N.V. Organon, Kloosterstraat 6, Netherlands)] was introduced from stimulation Day 6 when serum estradiol levels were above approximately 200 pg/mL, or delayed in cases of lower estradiol levels, according to follicular growth and hormonal monitoring. The presence of at least four follicles with a diameter of more than 15 mm combined with a serum level of E_2_ more than 150–200 pg/mL per follicle of more than 15 mm in diameter allowed us to trigger ovulation 36 h before follicular puncture using a GnRH agonist [DECAPEPTYL 0.2 mg (Ipsen, Paris, France)] or recombinant hCG [OVITRELLE 250 *μ*g (Merck Europe B.V., Amsterdam, Netherlands)]. The number of expected oocytes was defined as the number of follicles measuring at least 15 mm in diameter on the day of triggering.

Oocyte retrieval was performed by transvaginal ultrasound‐guided follicular aspiration, under either general anesthesia or local anesthesia injected into the vaginal fornices, depending on the patient’s preference. The follicular fluid was directly deposited in tubes containing IVF medium (CooperSurgical, Versailles, France) in an incubator at 37°C 5% CO_2_. Mature (metaphase II) and immature (germinal vesicle or germinal vesicle breakdown [GVBD]) oocytes were cryopreserved. Oocytes were mechanically and enzymatically treated with hyaluronidase (Syn Vitro Hyadase, Origio, Versailles, France) to remove the follicular cells from the corona radiata and to assess their maturity stage (Synvitro Hyadase kit, CooperMedical, Trumbull, United States). Oocyte vitrification was performed with the Vit Kit‐Freeze NX medium (FujiFilm Irvine Scientific) and high safety straws (CBS, L’Aigle, France). Straws were sealed, plunged, and stored into liquid nitrogen until use.

### 2.3. Statistical Analysis

Statistical analyses were performed using GraphPad Prism version 8.00 (GraphPad Software, La Jolla, CA, United States). Quantitative variables were described using mean, standard deviation, minimum, and maximum values, and qualitative variables using frequencies and percentages. The main descriptive analyses of women’s sociodemographic characteristics, motivations, fertility‐related knowledge, and POC cycle outcomes were considered pre‐specified. Subgroup comparisons and correlation analyses between available clinical and biological parameters and COH or oocyte retrieval outcomes were considered exploratory.

For quantitative variables, comparisons between groups were performed using Student’s *t*‐test or analysis of variance for parametric variables, and the Kruskal–Wallis test for non‐parametric variables. Subgroup comparisons included women who initiated COH versus those who did not, women aged < 35 years versus ≥ 35 years, and women with AMH levels < 1.1 ng/mL versus ≥ 1.1 ng/mL. Correlation analyses were conducted using Spearman’s rank correlation coefficient. The number of expected oocytes at triggering and the number of retrieved oocytes were compared using a paired Student’s *t*‐test. The oocyte recovery rate was defined as the ratio of retrieved to expected oocytes. Because some women underwent more than one COH cycle, analyses were first performed per cycle and then repeated at the patient level using a repeated‐measures ANOVA to account for within‐patient repeated observations. No adjustment for multiple testing was applied because subgroup and correlation analyses were exploratory; therefore, these findings were interpreted as exploratory associations. All statistical tests were two‐sided, and a *p* value < 0.05 was considered statistically significant.

### 2.4. Ethics Statement

This non‐interventional study complies with regulatory requirements related to the current French bioethics law on non‐medical fertility preservation. Informed consent was not required due to the retrospective design and the use of pre‐existing biological and clinical data, which were anonymized after collection, in accordance with the provisions of French non‐Jardé law. The study was registered with the Clinical Research Delegation of Rouen University Hospital under the reference number 2023/0133/OB1163 and received approval from the Institutional Review Board for Non‐Interventional Research (n°E2024‐66, date of approval August 30, 2024; CERDE) of Rouen University Hospital.

## 3. Results

### 3.1. Description of the Pre‐Treatment Process

**Figure 1:**
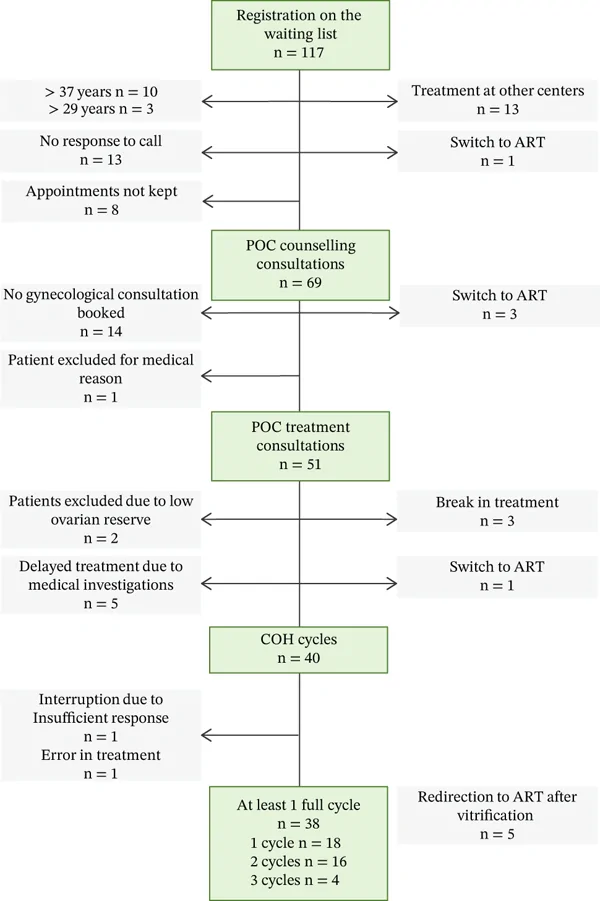
Flow chart of the study CECOS: Centre de conservation des œufs et du sperme; COH: Controlled Ovarian Hyperstimulation; *n*: number.

During the study period, 117 women contacted the Reproductive Biology Laboratory‐CECOS at Rouen Normandy University Hospital to be placed on the waiting list for POC (Figure 1). The mean age at registration was 34.7 ± 2.6 years (22.9–39.5), with 50.4% (*n* = 59/117) of women aged between 35 and 37 years. Most women resided in the Normandy region (54.6%, *n* = 64/117), while 42% (*n* = 49/117) came from the Paris region. Thirteen patients (11.1%, *n* = 13/117) who did not meet the legal age criteria were excluded from further evaluation (Figure 1). Overall, 65.8% (*n* = 77/117) appointments were booked and 69 patients attended their appointments (89.6%, *n* = 69/77) (Figure 1) and the time between registration and consultation appointment was 55.1 ± 43.75 days (0–214).

### 3.2. Personal Situation, Motivation, and Knowledge About POC

Women’s sociodemographic, reproductive, and professional characteristics are presented in Table 1. Briefly, the mean age at POC counselling consultation was 34.6 ± 1.9 years (29.6–37), with 43.5% (*n* = 30/69) of patients aged between 35 and 37 years. Most patients were single (**Q2**, 55%, *n* = 38/69) and heterosexual (**Q3**, 93%, *n* = 64/69). A current desire for pregnancy was reported by 42% (**Q10**, *n* = 29/69). Among those not currently wishing to conceive, pregnancy was considered as a possibility within the next 3.0 ± 1.3 years (range: 1.5–5.0). Almost all patients (**Q13**, 96%, *n* = 66/69) had completed higher education, with 86% (**Q15**, *n* = 59/69) having at least a Bachelor’s degree. One out of three patients (29%, *n* = 20/69) worked in the medical field and 70% (**Q16**, *n* = 48/69) reported having achieved their professional goal. Among the 21 women who had not yet achieved this goal, 38% (**Q17**, *n* = 8/21) considered essential to reach this latter before having a child (Table 1).

The proportion of patients who attended the POC counselling consultation on their own initiative was comparable with that of patients referred by a healthcare professional (**Q18**, 45%, *n* = 31/69 vs. 46%, *n* = 32/69, respectively). A majority of patients were informed by people close to them (**Q19**, 48%, *n* = 33/69), internet and social media (38%, *n* = 26/69), or the medical community (33%, *n* = 23/69).

Motivations, sources of information, knowledge, perceptions, and future intentions regarding POC are presented in Table 2.The principal motivations for undergoing POC (Q21) included awareness of age‐related decline in ovarian reserve (70%, *n* = 48/69), being single (48%, *n* = 33/69), and feeling pressure from the biological clock (29%, *n* = 20/69). Very few patients (**Q24**, *n* = 2) reported receiving encouragement from their employer regarding this process, and none of them received financial compensation (Table 2).

Only 7% of women (**Q27**, *n* = 5/69) believed that POC is a “100% guarantee” of having a child later. However, more than half of the respondents did not consider POC a medically risky procedure (**Q28**, 51%, *n* = 35/69). At the POC counselling consultation, 17% (**Q29**, *n* = 12/69) of patients were unaware that the entire POC process (treatment, retrieval, technique) is covered by the French health insurance; 29% of them (**Q30**, *n* = 20/69) stated that they would not have pursued this process without financial coverage. Most women planned to use their cryopreserved oocytes for a future parenting project only within the context of a couple (**Q31**, 59%, *n* = 41/69). Finally, if they did not need to use their oocytes for their own parenting project, 81% of women (**Q32**, *n* = 56/69) expressed willingness to donate them to couples, single women, or for research purposes (Table 2).

### 3.3. Clinical Characteristics at the Fertility Counselling Consultation

The majority of women were not using contraception (55%, *n* = 38/69), and nearly 27% (*n* = 18/69) reported a gynecological and/or obstetrical history, including voluntary abortion (14.5%, *n* = 10/69), miscarriage (4.3%, *n* = 3/69), endometriosis (7.2%, *n* = 5/69), polycystic ovary syndrome (2.9%, *n* = 2/69), and *Chlamydia trachomatis* genital infection (1.4%, *n* = 1/69). Additionally, the average body mass index (BMI) was 23.7 ± 4.5 kg/m^2^ (16.7–34.7). Following the POC counselling consultation, 81% of patients (*n* = 56/69) underwent ovarian reserve assessment. The mean AMH serum level was 2.1 ± 1.8 ng/mL (range: 0.1–8.3), the mean FSH serum level was 7.1 ± 2.6 IU/L (range: 5.5–18), and the mean AFC was 15.5 ± 10 (range: 2–49). There were no significant differences in BMI, AMH, FSH, or AFC between patients aged ≤ 35 years and those aged > 35 years (*p* = 0.3; *p* = 0.8; *p* = 0.9; and *p* = 0.9, respectively).

Finally, 74% of patients (*n* = 51/69) continued their care and attended a second consultation to confirm the feasibility of POC and determine the appropriate COH protocol (Figure 1). The average time between the POC counselling consultation and the second consultation was 80.2 ± 44.2 days (range: 13–223).

### 3.4. Characteristics of Women Receiving POC

Overall, 58% of patients (*n* = 40/69) underwent COH treatment for POC (Figure 1). The average time between registration for POC counselling consultation and the first oocyte retrieval was 194.1 ± 73.5 days (85–458). Two patients were excluded by the ART center’s multidisciplinary medical meeting due to very low ovarian reserve. Treatment was postponed for five patients pending additional medical evaluations. Three patients chose to temporarily pause their care pathway for personal or professional reasons, and one patient opted to pursue ART with sperm donation. Among women who attended the POC counselling consultation, baseline characteristics were compared between those who initiated COH and those who did not. No significant differences were observed between the two groups regarding age, relationship status, educational level, or fertility‐related knowledge (*p* = 0.9454; *p* = 0.1454; *p* = 0.5072, and *p* = 0.1495, respectively).

Of the 40 patients who underwent treatment, 95% (*n* = 38/40) completed at least one full cycle of ovarian stimulation leading to follicular puncture. A total of 65 COH cycles were initiated, and 62 complete cycles were performed. Among the patients, 47% (*n* = 18/38) underwent a single cycle, 42% (*n* = 16/38) underwent two cycles, and 11% (*n* = 4/38) received three cycles. One patient discontinued after the first COH cycle due to inadequate response, one patient was suspended due to a treatment error, and one patient was stopped due to premature ovulation, but she subsequently underwent additional cycles (Figure 1).

The mean age of these patients was 34.7 ± 1.6 years (range: 31.2–36.8), with 52.5% (*n* = 21/40) aged between 35 and 37 years. Twelve patients (30%, *n* = 12/40) had an AMH serum level below 1.1 ng/mL. No significant differences in BMI, AMH serum level, and AFC were observed between patients aged under and over 35 years (*p* = 0.2, *p* = 0.6, and *p* = 0.6, respectively). Similarly, no correlation was found between age and BMI, AMH level or AFC (r = 0.2, *p* = 0.2, r = −0.03, *p* = 0.9; r = −0.004, *p* = 0.98, respectively). However, a strong positive correlation was observed between AMH level and AFC (r = 0.8, *p* < 0.0001), and AMH level was significantly lower in patients with a higher BMI (r = −0.4, *p* = 0.02).

### 3.5. COH Cycles and Ovarian Puncture Outcomes

On average, each woman underwent 1.5 ± 0.7 (range: 0−3) complete COH cycles. Among these cycles, 85.4% (*n* = 53/62) followed antagonist protocols, of which 85% (*n* = 45/53) involved an estrogen‐progestin contraceptive pill, 9% (*n* = 5/53) used PROVAMES (Norgine SAS, Rueil‐Malmaison, France), and 6% (*n* = 3/53) used a microprogestin or progestin‐only pill. Additionally, 14.5% (*n* = 9/62) of the cycles followed a progestin‐primed ovarian stimulation (PPOS) protocol (Table 3).

**Table 3:** COH characteristics and outcomes per cycle, according to the type of COH protocol used.

	Total *n* = 62	Antagonist—micro/progestins *n* = 3	Antagonist—EPC *n* = 45	Antagonist—PROVAMES *n* = 5	PPOS *n* = 9	*p*
	Mean ± SD (range: min‐max)					
Age at ovarian puncture (years)	35.3 ± 2.2	36 ± 2	35.1 ± 2.4	36.5 ± 0.4	35 ± 2	0.5
	(31.3–37.5)	(33.7–37.2)	(23.1–37)	(35.9–36.8)	(31.3–37.5)	
BMI (kg/m^2^)	23.3 ± 4.5	21.2 ± 2.3	23.6 ± 4.7	26.7 ± 4.2	22.4 ± 4.3	0.3
	(16.7–33.1)	(18.5–22.5)	(16.7–33.1)	(21.4–32.1)	(17.9–30.4)	
AMH (ng/mL)	2.1 ± 1.7	1.6 ± 0.05	2.3 ± 2.1	1.9 ± 1	1.8 ± 1	0.99
	(0.3–8.3)	(1.5–1.6)	(0.3–8.3)	(0.6–3.17)	(0.3–4)	
AFC (*n*)	17.2 ± 10.4	18.3 ± 2.9	19.4 ± 13.1	14.2 ± 6.7	15.8 ± 5.7	0.8
	(2–49)	(15–20)	(2–49)	(7–24)	(9–27)	
FSH (IU/L)	6.8 ± 2.0	6.2 ± 2.2	7 ± 2	7.2 ± 3.1	5.5 ± 1	0.2
	(3.7–12.5)	(3.8–8)	(3.7–12.5)	(4.7–12.5)	(3.8–6.9)	
Starting dose (IU)	279.2 ± 67.3	300 ± 75	270.5 ± 63.1	287.5 ± 53.1	294.4 ± 79	0.8
	(150–450)	(225–375)	(150–450)	(250–375)	(200–375)	
Trigger dose (IU)	277.6 ± 90.4	283.3 ± 52.1	272.1 ± 85.6	277.5 ± 65.2	272.2 ± 87	0.98
	(75–450)	(225–325)	(75–450)	(200–375)	(150–375)	
Total dose (IU)	3193.3 ± 112.8	3033 ± 689.8	3204 ± 1187	3065 ± 413.7	2986 ± 721.3	0.99
	(1225–6300)	(2250–3550)	(1225–6300)	(2625–3750)	(1750–3750)	
Antagonist introduction (days)	6.9 ± 2.5	5.7 ± 0.6	7.2 ± 1.5	6.2 ± 1.1	6 ± 0	**0.04**
	(5–12)	(5–6)	(5–12)	(5–8)	(6–6)	
E_2_ at trigger (pg/mL)	1290 ± 721.9	1121 ± 945.4	1298 ± 650.8	1887 ± 1019	977.8 ± 677.7	0.2
	(229–3519)	(428–2198)	(229–2937)	(1103–3519)	(357–2353)	
Trigger (days)	12.6 ± 1.9	11.7 ± 0.6	12.9 ± 2.075	11.8 ± 1.1	11.7 ± 1	0.1
	(10–19)	(11–12)	(10–19)	(11–13)	(11–14)	
Collected oocytes (*n*)	9.1 ± 6.8	6 ± 3	9.6 ± 7.5	7.8 ± 3.2	8.8 ± 5	1
	(0–36)	(3–9)	(0–36)	(4–12)	(0–19)	
Cryopreserved oocytes (*n*)	8.3 ± 6	5.7 ± 2.5	8.6 ± 6.5	7 ± 3.5	8.6 ± 5	0.3
	(0–31)	(3–8)	(0–31)	(3–12)	(0–19)	
Mature oocytes (*n*)	7.3 ± 5.3	5.7 ± 2.5	7.5 ± 5.8	6.2 ± 3.1	7.7 ± 4.7	0.9
	(0–28)	(3–8)	(0–28)	(3–3)	(0–18)	
Maturation rate (%)	80 ± 17.4	96.3 ± 6.4	79.5 ± 19.7	77.3 ± 9.4	87.6 ± 13.9	0.2
	(36.4–100)	(88.9–100)	(36.4–100)	(66.7–90)	(63.7–100)	

*Note:* Boldface indicates that *p* < 0.05 was considered significant.

Abbreviations: AFC: antral follicle count; AMH: anti‐Müllerian hormone; BMI: body mass index; COH: controlled ovarian hyperstimulation; EPC: estrogen–progestin contraception; E_2_: Estradiol; FSH: follicle stimulating hormone; min: minimum; max: maximum; *n*: number; PPOS: progestin‐primed ovarian stimulation; SD: standard deviation.

The majority of COH cycles (84%, *n* = 52/62) were stimulated with recombinant FSH alpha (FSHr *α*), including GONAL‐F (*n* = 37) and BEMFOLA (*n* = 15). This was followed by 13% (*n* = 8/62) using recombinant FSH beta (FSHr *β*), and 3% (*n* = 2/62) using a combination of FSHr *α* and recombinant LH alpha (LHr *α*). Almost all ovulation triggerings were performed using a 0.2 mg dose of a GnRH agonist (97%, *n* = 60/62), and 82% (*n* = 51/62) of the oocyte retrievals were carried out under general anesthesia.

A total of 572 oocytes were retrieved and 539 oocytes were cryopreserved, of which 474 were mature metaphase II oocytes. When considering all COH cycles performed per woman, the mean number of retrieved oocytes was 15.1 ± 11.8 per woman (range: 5–58). The mean number of cryopreserved oocytes was 13.8 ± 10.6 per woman (range: 4–50), including 12.1 ± 8.8 mature metaphase II oocytes per woman (range: 3–46). Empty punctures occurred in 3.2% of cases (*n* = 2/62). Following oocyte retrieval, one patient developed ovarian hyperstimulation syndrome, and one procedure was complicated by an upper genital tract infection.

Two subgroups were defined based on patient age: strictly under 35 years and 35 years or older. Only the results from the first COH cycle were considered. The starting gonadotropin dose was significantly higher in patients over 35 years (239.5 ± 48.8 IU, range: 150–375 vs. 276.8 ± 53.1 IU, range: 200–375; *p* = 0.02). However, no significant differences were observed in the triggering of gonadotropins, duration of stimulation, or E_2_ levels at trigger. Likewise, no differences were found in the number of oocytes retrieved or cryopreserved, nor in the oocyte maturation rate per cycle.

Moreover, two subgroups of patients were determined based on the Bologna criteria, with initial AMH levels strictly below or above 1.1 ng/mL [50]. Once again, only the results of the first ovarian puncture were considered. The starting, triggering, and total gonadotropin doses were significantly higher in patients with an AMH serum level strictly below 1.1 ng/mL (307.5 IU ± 51 (range: 250–375) vs. 238.8 IU ± 38 (range: 150–300), *p* < 0.0001; 330.6 IU ± 117 (range: 250–375) vs. 231.3 IU ± 58 (range: 112.5–375), *p* < 0.0001; 3419 IU ± 824 (range: 2100–4875) vs. 2647.3 IU ± 710 (range: 1575–4650), *p* = 0.002, respectively). However, neither the E_2_ levels nor the trigger day differed between the two groups (*p* = 0.4 and *p* = 0.4, respectively). Finally, the number of retrieved, mature, or cryopreserved oocytes was lower in patients with an AMH serum level strictly below 1.1 ng/mL (6.3 ± 1.8 (range: 3–8) vs. 11.3 ± 7 (range: 3–36), *p* = 0.009, *p* = 0.006; 4.9 ± 1.9 (range: 2–8) vs. 9.4 ± 5.6 (range: 3–28), *p* = 0.001 and 5.8 ± 2.2 (range: 2–8) vs. 10.3 ± 6.3 (range: 3–31), respectively).

### 3.6. Analysis per Cycle of COH and Ovarian Puncture Outcomes

The results for factors influencing COH are presented in Table 4. AMH level as well as AFC were inversely correlated with the starting, triggering, and total doses of gonadotropins (*r* = −0.6, *p* < 0.0001; *r* = −0.7, *p* < 0.0001; *r* = −0.4, *p* = 0.0006 for AMH and *r* = −0.5, *p* < 0.0001; *r* = −0.5, *p* < 0.0001; *r* = −0.3, *p* = 0.01 for AFC, respectively). Higher BMI was associated with higher starting, triggering, and total doses of gonadotropins (*r* = 0.4, *p* = 0.001; *r* = 0.5, *p* = 0.0003; *r* = 0.4, *p* = 0.005, respectively). Age did not impact the COH cycle data, except for a higher E_2_ level at triggering (*r* = 0.3, *p* = 0.01). Additionally, basal FSH serum level at the POC counselling consultation was not predictive of stimulation characteristics, except for a delayed introduction of the antagonist in patients with higher FSH levels (r = 0.3, *p* = 0.04) (Table 4).

**Table 4:** Correlations between intrinsic patient characteristics, COH parameters, and ovarian puncture outcomes, per cycle.

	Age (years)	AMH (ng/mL)	AFC	FSH (IU/L)	BMI (kg/m^2^)	Starting dose (IU)	E_2_ at trigger (pg/mL)
AMH (ng/mL)	*p* = 0.7						
	*r* = 0.06						
AFC (*n*)	*p* = 0.3	**p** < 0.0001					
	*r* = 0.2	*r* = 0.8					
FSH (IU/L)	*p* = 0.1	*p* = 0.6	*p* = 0.3				
	*r* = 0.3	*r* = 0.07	*r* = 0.2				
BMI (kg/m^2^)	*p* = 0.2	**p** = 0.01	*p* = 0.5	*p* = 0.5			
	*r* = 0.2	*r* = −0.3	*r* = −0.09	*r* = 0.1			
Starting dose (IU)	*p* = 0.9	**p** < 0.0001	**p** < 0.0001	*p* = 0.6	**p** = 0.001		
	*r* = −0.02	*r* = −0.6	*r* = −0.5	*r* = 0.1	*r* = 0.4		
Trigger dose (IU)	*p* = 0.2	**p** = <0.0001	**p** < 0.0001	*p* = 0.6	**p** = 0.0003	**p** < 0.0001	**p** = 0.004
	*r* = −0.2	*r* = −0.7	*r* = −0.5	*r* = 0.1	*r* = 0.5	*r* = 0.9	*r* = −0.4
Total dose (IU)	*p* = 0.1	**p** = 0.0006	**p** = 0.01	*p* = 0.4	**p** = 0.0045	**p** < 0.0001	*p* = 0.1
	*r* = −0.2	*r* = −0.4	*r* = −0.3	*r* = 0.1	*r* = 0.4	*r* = 0.7	*r* = −0.2
Antagonist introduction (days)	*p* = 0.3	*p* = 0.57	*p* = 0.4	**p** = 0.04	*p* = 0.3	*p* = 0.6	*p* = 0.3
	*r* = −0.2	*r* = 0.08	*r* = 0.1	*r* = 0.3	*r* = 0.2	*r* = 0.1	*r* = −0.1
E_2_ at trigger (Pg/mL)	**p** = 0.01	*p* = 0.1	*p* = 0.07	*p* = 0.6	*p* = 0.9	*p* = 0.2	
	*r* = 0.3	*r* = 0.2	*r* = 0.2	*r* = 0.1	*r* = −0.01	*r* = −0.2	
Trigger day	*p* = 0.2	*p* = 0.7	*p* = 0.8	*p* = 0.06	*p* = 0.9	*p* = 0.8	*p* = 0.4
	*r* = −0.2	*r* = 0.05	*r* = 0.04	*r* = 0.3	*r* = −0.01	*r* = 0.04	*r* = −0.1
Collected oocytes (*n*)	*p* = 0.2	**p** < 0.0001	**p** < 0.0001	*p* = 0.2	*p* = 0.1	**p** = 0.0004	**p** = 0.0008
	*r* = 0.2	*r* = 0.6	*r* = 0.6	*r* = −0.2	*r* = −0.2	*r* = −0.4	*r* = 0.4
Cryopreserved oocytes (*n*)	*p* = 0.3	**p** < 0.0001	**p** < 0.0001	*p* = 0.2	*p* = 0.1	**p** < 0.0001	**p** = 0.0008
	*r* = 0.1	*r* = 0.6	*r* = 0.6	*r* = −0.2	*r* = −0.2	*r* = −0.5	*r* = 0.4
Atretic oocytes (*n*)	*p* = 0.5	**p** = 0.02	**p** = 0.0003	*p* = 0.8	*p* = 0.6	*p* = 0.2	*p* = 0.7
	*r* = 0.1	*r* = 0.3	*r* = 0.5	*r* = −0.04	*r* = −0.1	*r* = −0.2	*r* = 0.05
EZP (*n*)	*p* = 0.07	*p* = 0.1	**p** = 0.04	*p* = 0.3	*p* = 0.9	*p* = 0.5	**p** = 0.01
	*r* = 0.2	*r* = 0.2	*r* = 0.3	*r* = −0.2	*r* = 0.02	*r* = −0.1	*r* = 0.3
GV (*n*)	*p* = 0.2	*p* = 0.1	**p** = 0.003	*p* = 0.6	*p* = 0.1	*p* = 0.5	*p* = 0.95
	*r* = 0.2	*r* = 0.2	*r* = 0.4	*r* = −0.1	*r* = 0.2	*r* = −0.1	*r* = 0.008
GVBD (*n*)	*p* = 0.4	*p* = 0.1	**p** = 0.0004	*p* = 0.2	*p* = 0.3	*p* = 0.5	*p* = 0.4
	*r* = 0.1	*r* = 0.2	*r* = 0.4	*r* = −0.2	*r* = 0.1	*r* = −0.1	*r* = 0.1
Mature oocytes (*n*)	*p* = 0.4	**p** < 0.0001	**p** < 0.0001	*p* = 0.3	**p** = 0.02	**p** < 0.0001	*p* = 0.0004
	*r* = 0.1	*r* = 0.7	*r* = 0.6	*r* = −0.2	*r* = −0.3	*r* = −0.5	*r* = 0.5
Maturation rate (%)	*p* = 0.2	*p* = 0.1	*p* = 0.4	*p* = 0.6	**p** = 0.04	**p** = 0.002	*p* = 0.5
	*r* = −0.2	*r* = 0.2	*r* = 0.1	*r* = −0.1	*r* = −0.3	*r* = −0.4	*r* = 0.1

*Note:* Boldface indicates that *p* < 0.05 was considered significant.

Abbreviations: AFC: antral follicle count; AMH: anti‐Müllerian hormone; BMI: body mass index; COH: controlled ovarian hyperstimulation; EZP: Empty Zona Pellucida; E_2_: estradiol; FSH: follicle stimulating hormone; GV: germinal vesicle; GVBD: germinal vesicle breakdown.

Moreover, AMH level was positively correlated with the number of retrieved, cryopreserved, mature, and atretic oocytes (*r* = 0.6, *p* < 0.0001; *r* = 0.7, *p* < 0.0001 and *r* = 0.3, *p* = 0.02, respectively). Higher AFC was associated with greater numbers of retrieved, mature, GVBD, GV, empty zona pellucida, and atretic oocytes (*r* = 0.6; *p* < 0.0001; *r* = 0.6; *p* < 0.0001; *r* = 0.4; *p* = 0.0004; *r* = 0.4; *p* = 0.003; *r* = 0.3; *p* 0.04 and *r* = 0.5; *p* = 0.0003, respectively). Conversely, higher starting doses of gonadotropins were associated with lower numbers of retrieved oocytes, mature cryopreserved oocytes, and total cryopreserved oocytes. (*r* = −0.44; *p* = 0.0004, and *r* = −0.52; *p* < 0.0001, *r* = −0.48; *p* < 0.0001, respectively). No correlation was found between age or FSH levels and the outcomes of oocyte retrieval (Table 4). Notably, the oocyte maturation rate per cycle was inversely correlated with both higher BMI and higher starting doses of gonadotropins (*r* = −0.27; *p* = 0.04 and *r* = 0.09; *p* = 0.002) (Table 4). To determine whether the association between BMI and oocyte maturation could reflect a technical retrieval bias, the number of retrieved oocytes was compared with the number of expected oocytes at triggering. No significant difference was observed between the numbers of expected and retrieved oocytes (10.4 ± 5.6 [range: 4−30] vs. 9.2 ± 6.9 [range: 0−36], *p* = 0.089), and BMI was not significantly correlated with the number of expected oocytes at triggering, the number of retrieved oocytes, or the oocyte recovery rate, defined as the ratio of retrieved to expected oocytes (*r* = −0.25, *p* = 0.054; *r* = −0.15, *p* = 0.27; *r* = 0.03, *p* = 0.82, respectively).

### 3.7. Repeated Measures Analysis

The repeated‐measures analyses by woman, accounting for the variable number of cycles per participant, showed consistent results for COH parameters (Table 5). AMH levels were inversely correlated with the starting, triggering, and total gonadotropin doses administered (*r* = −0.7, *p* < 0.001; *r* = −0.7, *p* < 0.001; *r* = −0.4, *p* = 0.01, respectively). Similarly, higher AFC was associated with lower starting and triggering gonadotropin doses (*r* = −0.5, *p* = 0.001; *r* = −0.5, *p* = 0.002). Additionally, higher BMI was correlated with higher starting, triggering, and total received gonadotropin doses (*r* = 0.48, *p* = 0.002; *r* = 0.49, *p* = 0.002; *r* = 0.36, *p* = 0.025). Furthermore, age did not impact ovarian stimulation parameters, except for a higher E_2_ level at triggering in older patients (*r* = 0.34, *p* = 0.04) (Table 5).

**Table 5:** Correlations between intrinsic patient characteristics, COH parameters, and ovarian puncture outcomes per patient, following repeated measures analysis.

	Age	AMH (ng/mL)	AFC (*n*)	BMI (kg/m^2^)
Starting dose (IU)	*p* = 0.7	**p** < 0.0001	**p** = 0.0009	**p** = 0.002
	*r* = 0.1	*r* = −0.7	*r* = −0.5	*r* = 0.5
Trigger dose (IU)	*p* = 0.7	**p** < 0.0001	**p** = 0.002	**p** = 0.002
	*r* = −0.1	*r* = −0.7	*r* = −0.5	*r* = 0.5
Total dose (IU)	*p* = 0.6	**p** = 0.01	*p* = 0.14	**p** = 0.03
	*r* = −0.1	*r* = −0.4	*r* = −0.2	*r* = 0.4
Antagonist introduction (days)	*p* = 0.5	*p* = 0.6	*p* = 0.3	*p* = 0.2
	*r* = −0.1	*r* = 0.1	*r* = 0.2	*r* = 0.2
E_2_ at trigger(pg/mL)	**p** = 0.04	*p* = 0.2	*p* = 0.2	*p* = 0.8
	*r* = 0.34	*r* = 0.2	*r* = 0.2	*r* = −0.04
Trigger(days)	*p* = 0.6	*p* = 0.99	*p* = 0.8	*p* = 0.9
	*r* = −0.1	*r* = 0.002	*r* = 0.04	*r* = −0.03
Collected oocytes (*n*)	*p* = 0.99	**p** < 0.0001	**p** = 0.0002	*p* = 0.1
	*r* = −0.0003	*r* = 0.6	*r* = 0.6	*r* = −0.3
Cryopreserved oocytes (*n*)	*p* = 0.8	**p** < 0.0001	**p** = 0.0001	*p* = 0.1
	*r* = −0.04	*r* = 0.6	*r* = 0.6	*r* = −0.3
Atretic oocytes (*n*)	*p* = 0.7	**p** = 0.02	**p** = 0.004	*p* = 0.4
	*r* = 0.1	*r* = 0.4	**r** = 0.5	*r* = −0.2
EZP (*n*)	*p* = 0.1	*p* = 0.3	*p* = 0.4	*p* = 0.99
	*r* = 0.3	*r* = 0.2	*r* = 0.2	*r* = −0.001
GV (*n*)	*p* = 0.3	*p* = 0.4	*p* = 0.07	**p** = 0.045
	*r* = 0.2	*r* = 0.1	*r* = 0.3	*r* = 0.3
GVBD (*n*)	*p* = 0.9	*p* = 0.2	**p** = 0.0024	*p* = 0.5
	*r* = 0.02	*r* = 0.2	*r* = 0.5	*r* = 0.1
Mature oocytes (*n*)	*p* = 0.7	**p** < 0.0001	**p** = 0.0004	**p** = 0.04
	*r* = −0.1	*r* = 0.7	*r* = 0.6	*r* = −0.4

*Note:* Boldface indicates that *p* < 0.05 was considered significant.

Abbreviations: AFC: antral follicle count; AMH: anti‐Müllerian hormone; BMI: body mass index; COH: controlled ovarian hyperstimulation; E_2_: estrogen levels; EZP: Empty Zona Pellucida; FSH: follicle stimulating hormone; GV: germinal vesicle; GVBD: germinal vesicle breakdown.

Regarding oocyte retrieval outcomes, repeated‐measures analyses by patient showed a positive correlation between AMH level or AFC and the number of retrieved (*r* = 0.63, *p* < 0.001 and *r* = 0.58, *p* = 0.0002), cryopreserved (*r* = 0.63, *p* < 0.0001 and *r* = 0.59, *p* = 0.0001), mature (*r* = 0.66, *p* < 0.001 and *r* = 0.55, *p* = 0.0004), and atretic oocytes (*r* = 0.37, *p* = 0.02 and *r* = 0.46, *p* = 0.004) and the numbers of GVBD retrieved also increases with AFC (*r* = 0.48; *p* = 0.0024). Higher BMI was correlated with a lower number of mature oocytes and a higher number of GV (*r* = −0.35, *p* = 0.04 and *r* = 0.33, *p* = 0.045). However, no correlation was found between the patient’s age at the time of oocyte retrieval and COH outcomes (Table 5).

## 4. Discussion

The preservation of fertility for non‐medical reasons, commonly referred as “social reasons” or POC, has been authorized in France since August 2, 2021 following the revision of Bioethics Law No. 2021‐10170 [11] with implementation through Decree No. 2021‐1933 dated December 30, 2021 [9, 10]. Our study examined the population of women who benefited from POC as well as the outcomes of this procedure, from the implementation of the decree in December 2021 until December 2022 at Rouen University Hospital. Over the course of nearly a year, we have been monitoring this new ART activity and attempted to compare our data with those from countries where this practice has been established for several years.

### 4.1. Profile of Women Seeking POC in the New French Framework

During this period, over 40% of the patients seeking POC at our institution were from the Paris region. These patients chose our center due to the prolonged waiting times experienced in Parisian centers since the implementation of the decree. The average age of these women was 35 years, which is younger than in foreign centers where the average age was around 37 years [16, 18–20, 22, 23, 25–36, 38–40, 46]. This age difference is intrinsically linked to French regulations, which restrict access to POC up to the age of 37 [9, 10, 51]. According to the proposed questionnaire, our patients were mostly single, heterosexual, and expressed a desire for pregnancy either immediately or within the next 3 years. They were more educated than the general French population, with 85% having an educational level equal to or higher than a Bachelor’s degree, compared with only 30%–39% of women aged 24–45 years having a similar level of education in 2020, according to INSEE (French National Institute of Statistics and Economic Studies) [52].

### 4.2. Motivations, Knowledge, and Perceptions of POC

The motivations of our patients were similar to those described in other countries, with the main reasons for undertaking this process being awareness of the decline in ovarian reserve with age and being single [22, 25, 26, 29, 30, 33, 35, 36]. These women primarily intended to reuse their cryopreserved oocytes when in a couple, and 56% indicated a potential willingness to donate oocytes to other women if not used themselves. This result differed from international literature, which reports lower rates of potential oocyte donation to other women, ranging from 4.8% to 31% [16, 22, 25, 30, 33, 35]. However, our findings are consistent with a recent study conducted in France, in which 48% of participants indicated they would consider donating their unused oocytes to other women [51].

Our patients tended to overestimate the age limit for achieving satisfactory OC, extending it to nearly 38 years. However, literature reports that the number of cryopreserved oocytes per woman is almost halved after the age of 35, with 9.6 oocytes at ages 31–35 vs. 5.63 at ages 36–40 [41]. Additionally, 7% of women attending POC counselling consultations viewed OC as a “100% guarantee” of having a child in the future. Another important finding is that the majority of patients did not perceive POC as a medically risky procedure, despite the cumulative risks associated with hormonal ovarian stimulation, complications related to oocyte retrieval, and, where applicable, general anesthesia. These findings highlight the biased and idealized perception that this practice may hold in the public’s mind, underlining the importance of delivering accurate information during counselling sessions and through public communication by the French Biomedicine Agency.

### 4.3. Early Clinical Outcomes of COH Cycles

From a medical standpoint, the patients were generally healthy women with good lifestyle habits and regular gynecological follow‐up. Some patients underwent their ovarian reserve evaluation while using hormonal contraception, which may have influenced AMH interpretation. Indeed, the literature indicates that oral contraception induces a 29.8% decrease in serum AMH levels and potentially leading to an underestimation of ovarian reserve [53]. Nevertheless, in women without an immediate desire for pregnancy, interrupting contraception for 2–3 months solely to optimize ovarian reserve assessment may not be acceptable from a patient‐centered perspective, particularly given the risk of disrupting contraceptive choices and delaying access to care.

Overall, just under half of the registered patients underwent COH. For these patients, our approach aimed to maximize the number of cryopreserved oocytes whenever possible. Therefore, in addition to mature oocytes, we also vitrified oocytes at the GVBD and GV stages, given the potential future role of in vitro maturation techniques in increasing the number of usable oocytes per patient [54, 55]. Patients were informed during counseling that these immature oocytes do not currently have the same established clinical value as mature oocytes but may become relevant depending on future advances in in vitro maturation.

### 4.4. Determinants of Oocyte Retrieval Outcomes

In our study, we found a positive correlation between ovarian reserve (AMH and AFC) and the number of cryopreserved oocytes. However, there was also an increase in the number of atretic oocytes as AMH and AFC levels rose. Additionally, an increase in AFC was specifically associated with an increase in the number of GV, GVBD, and empty zona pellucida. This is likely due to the collection of a greater total number of oocytes and a heterogeneous cohort of stimulated follicles. It is important to note that no statistical link was observed in our study between ovarian reserve markers and oocyte maturation rate. Similarly, Gambini et al. [56] examined the risk factors for poor oocyte retrieval and immaturity after triggering with a GnRH agonist. A significant association was found between lower AMH or AFC levels and a reduced number of retrieved oocytes (*p* = 0.04 and *p* = 0.016, respectively). However, these markers were not associated with a significant risk of oocyte immaturity (*p* = 0.18 and *p* = 0.35, respectively) [56].

In contrast to other international studies, no association was found between age and the outcomes of oocyte retrieval. This is likely due to the narrower age range at the time of retrieval, as mandated by French regulations (29–37 years), compared with studies conducted in other countries, which include broader age ranges and higher mean ages (36.9 years [23–43]; 37.2 years [≤ 25–≥ 40] or unspecified average [≤ 21–≥ 45]) [34, 39, 41]. Indeed, when focusing on the same age ranges, these studies also did not report any differences between patients under 35 years and those aged 35–38 years.

Among the other factors studied, higher BMI was associated with a lower number of mature oocytes and a reduced maturation rate. However, BMI was not associated with the number of expected oocytes at triggering, the total number of retrieved oocytes, or the oocyte recovery rate. These findings argue against a technical retrieval bias related to BMI. The association observed between higher BMI and reduced oocyte maturity may instead suggest an effect on oocyte competence. To our knowledge, this factor has not been specifically analyzed in previous studies on POC. However, similar findings have been reported in studies on OC for medical indications. Indeed, Lainas *et al*. [57] supported these findings, showing a significantly lower number of metaphase II oocytes in patients with a BMI > 25 kg/m^2^ (*p* = 0.029) after triggering with a GnRH agonist within the context of ART. Differences in drug bioavailability were likely to explain these observations and the authors suggested performing a double trigger to mitigate this risk [57]. Conversely, Donno *et al*. [58] found no difference in oocyte maturation based on BMI in a retrospective study of 5190 cycles of ART and medical fertility preservation, regardless of the type of trigger (GnRH agonist, hCG, or double trigger). This team suggests that BMI should not be a consideration in the choice of trigger [58]. Further studies on this matter, within the context of both societal and medical fertility preservation, are necessary.

We recorded only one complication due to ovarian hyperstimulation syndrome and noticed no significant difference between the starting and trigger doses of gonadotropins. Therefore, we can conclude that no overestimation of the FSH dose occurred at treatment initiation due to a potential underestimation of ovarian reserve in patients using hormonal contraception. Consequently, discontinuation of hormonal contraception does not appear to be necessary in the management of patients undergoing fertility preservation.

### 4.5. Influence of the French Legal and Reimbursement Framework

However, it is important to emphasize that the conditions for accessing POC abroad differ from those in France. In France, the number of POC cycles is primarily limited by intrinsic patient characteristics, such as age, poor ovarian response to COH, or medical contraindications. In contrast, abroad, financial constraints are likely to represent a major limiting factor in the repetition of cycles and the number of mature cryopreserved oocytes per woman.

To date, France is the only country where POC is fully covered by the national health insurance. In our study, 17% of women expected to pay medical fees in order to receive POC. In 2017, a descriptive study asking 407 French reproductive physicians about POC found that 86% wanted patients to be responsible for treatment costs [59]. Abroad, POC is fully financed by the patient, with the cost of reusing stored oocytes estimated between $10.000 and $35.000 [48]. Some foreign doctors question the relevance of proposing POC, given the considerable cost associated with this procedure [60], while reuse rates are low, and pregnancy rates are limited by the age of OC and reuse [34, 39]. Coverage by the French health system means that this approach can be introduced without worrying about the patient’s financial situation. Notably, 29% of patients at our center would not have undergone the procedure if it had been at their own expense. In the future, it will be crucial to evaluate the reuse of cryopreserved oocytes and the resulting pregnancy rates in our country.

Although public funding may reduce the direct financial barrier to POC, it does not necessarily ensure equal access for all women. Access to POC may still depend on awareness of the procedure, health literacy, referral by healthcare professionals, geographical proximity to an authorized ART center, and the ability to attend several consultations and monitoring visits. The high educational level observed in our cohort suggests that socioeconomic and informational gradients may persist despite reimbursement by the national health insurance system. Further studies are needed to determine whether publicly funded POC effectively reaches the broader population of women who may be interested in fertility preservation.

### 4.6. Strengths and Limitations

Our study has several strengths. First, although the analysis was retrospective, data were collected prospectively and in a standardized manner from the opening of the POC pathway following the implementation of Decree No. 2021‐1933 on December 30, 2021 [9, 10]. This allowed the creation of a structured cohort and limited heterogeneity in data collection. Second, our study provides data on the early implementation of POC in France by combining information on women’s sociodemographic profiles, motivations, fertility‐related knowledge, and early clinical outcomes. Finally, the detailed collection of clinical and biological parameters allowed us to explore associations between intrinsic patient characteristics, COH protocols, and oocyte vitrification outcomes.

However, our study has several limitations. First, this was a single‐center study with a limited sample size, although it reflects the real‐life activity of our center during the early implementation of POC in France. Second, follow‐up remains too short to assess the utilization of cryopreserved oocytes, pregnancy rates, or live birth outcomes. Therefore, our clinical results should be interpreted as early vitrification outcomes rather than reproductive outcomes.

Another limitation is the progressive attrition observed throughout the POC pathway. Although 117 women initially registered for POC, only 40 initiated COH treatment and 38 completed at least one full cycle. This proportion should not be interpreted as the treatment uptake rate among eligible and fully counselled women, because the initial group of 117 registered women included patients who were later found to be ineligible, did not attend or book appointments, could not be reached, or were redirected to ART before ovarian stimulation could be offered. Among women who attended the POC counselling consultation, no significant differences were observed between those who initiated COH and those who did not regarding age, relationship status, educational level, or fertility‐related knowledge. Nevertheless, the analysis of COH outcomes was restricted to women who proceeded to ovarian stimulation, and unmeasured factors, such as personal availability, motivation, logistical constraints, or readiness to engage in a time‐consuming medical pathway, may still have influenced continuation of care.

Finally, our cohort may not be fully representative of all women potentially interested in POC. Women who attended counselling were mostly highly educated and may have had greater access to information, healthcare professionals, and specialized ART pathways. This may introduce a selection bias and limit the generalizability of our findings to the broader population of women who may be interested in POC. Finally, motivations and fertility‐related knowledge were self‐reported during the counselling consultation and may therefore be subject to recall bias, reporting bias, or social desirability bias.

### 4.7. Perspectives

The progressive increase in POC requests observed during the study period also supports the need for continued monitoring. Between the first and last trimesters of the study, the average number of monthly registrations on the waiting list increased from 4.7 to 11.3, reflecting growing interest in POC after implementation of the French legal framework.

Whether the French legal and reimbursement framework will influence future oocyte utilization rates or reproductive outcomes remains unknown. Compared with countries where POC is self‐funded, public coverage and regulated access may affect both the decision to undergo POC and the conditions under which stored oocytes are later used. However, reuse rates, pregnancy rates, and live birth outcomes depend on multiple factors, including duration of follow‐up, age at OC and at reuse, access to ART services and legal conditions for using stored oocytes. Therefore, longer‐term follow‐up of French cohorts will be required before drawing conclusions regarding oocyte utilization, pregnancy rates, or live birth outcomes in this specific publicly funded setting [20, 33, 34, 39, 43–45, 47, 48].

POC may also raise questions regarding the future availability of unused cryopreserved oocytes for donation in France. In our cohort, 56% of women indicated that they would consider donating their unused oocytes to other women. However, whether these intentions will translate into actual donation remains unknown and will require specific follow‐up.

Finally, 12 patients from our cohort were redirected toward a pregnancy project during their care at our center (with or without ART). Beyond OC itself, the POC pathway may therefore represent an opportunity to provide individualized counselling on ovarian reserve, age‐related fertility decline, and realistic reproductive options. In some cases, this information may help women reconsider the timing of their reproductive plans or redirect them toward a more appropriate reproductive pathway.

## 5. Conclusion

In the context of increasing age at first childbirth and persistent gaps in awareness regarding age‐related fertility decline, POC may represent an additional reproductive option for women wishing to preserve future fertility. In this early single‐center French cohort, women seeking POC after implementation of the 2021 Bioethics Law were mostly highly educated and were mainly motivated by single status and awareness of age‐related fertility decline. Early vitrification outcomes were associated with ovarian reserve markers and BMI, whereas age was not associated with oocyte yield within the restricted age range allowed by French regulations. These findings provide preliminary real‐life data on the implementation of publicly funded POC in France. Longer‐term follow‐up is required to assess oocyte utilization, pregnancy and live birth outcomes, and the impact of this new practice on access to fertility preservation and future parenthood.

## Author Contributions

C.T. and R.L. conducted planned oocyte cryopreservation counselling consultation and collected the questionnaire responses. C.T., A.F., and N.R. contributed to the study conceptualization and design, supervision of the research and validation of the results and were responsible for data collection, processing, and manuscript writing. M.L., F.J., N.K. and R.L. contributed to the validation of the results, review and editing of the manuscript.

## Funding

No funding was received for this manuscript.

## Disclosure

All authors have read and approved the final version of the manuscript. Pr. Nathalie Rives, as corresponding author and manuscript guarantor, had full access to all of the data in this study and takes complete responsibility for the integrity of the data and the accuracy of the data analysis. Pr. Nathalie Rives, as manuscript guarantor, affirms that this manuscript is an honest, accurate, and transparent account of the study being reported; that no important aspects of the study have been omitted; and that any discrepancies from the study as planned have been explained.

## Conflicts of Interest

The authors declare no conflicts of interest.

## Supporting information


**Supporting Information** Additional supporting information can be found online in the Supporting Information section. File 1 STROBE_checklist_Taibi_JOP. This supplementary file contains the completed STROBE (Strengthening the Reporting of Observational Studies in Epidemiology) checklist for cohort studies. For each of the 22 STROBE items, the checklist indicates where the corresponding information is reported in the manuscript, with the relevant page number and a short note. It is provided to document the compliance of this single‐center retrospective observational study with the STROBE reporting guideline.

## Data Availability

The data that support the findings of this study are available from the corresponding author upon reasonable request. The data are not publicly available due to privacy and ethical restrictions related to patient information.
