# Supplementary material for: Planned Oocyte Cryopreservation Following the 2021 French Bioethics Law: Women’s Characteristics and Early Clinical Outcomes From a Single‐Center Observational Study
Source: J Pregnancy. 2026 Sep 6;2026:7642675. doi: 10.1155/jp/7642675 (PMC13547955; doi:10.1155/jp/7642675)
Supplement: Supplementary file 1 [file JP-2026-7642675-s001.docx]

STROBE Statement—Checklist of items that should be included in reports of ***cohort studies***

|  | Item No | Recommendation | Page No. | Reported / Notes |
| --- | --- | --- | --- | --- |
| **Title and abstract** | 1 | (*a*) Indicate the study’s design with a commonly used term in the title or the abstract | 1 | Title includes “...Early Clinical Outcomes from a Single-Center Observational Study”. |
|  |  | (*b*) Provide in the abstract an informative and balanced summary of what was done and what was found | 1–2 | Structured abstract (Background and Aims / Methods / Results / Conclusion): 117 registered, 69 attended, 40 initiated COH; AMH/AFC positive, BMI negative associations. |
| Introduction | | | | |
| Background/rationale | 2 | Explain the scientific background and rationale for the investigation being reported | 2–4 | Introduction §1–6: rising maternal age in France, age-related fertility decline, background on POC internationally and under the 2021 French Bioethics Law. |
| Objectives | 3 | State specific objectives, including any prespecified hypotheses | 4 (also p.1) | “The primary objective of our study was to describe… The secondary objectives were to report early clinical outcomes… and to explore associations…” (end of Introduction; restated in Abstract). |
| Methods | | | | |
| Study design | 4 | Present key elements of study design early in the paper | 4 | First sentence of Methods: “We conducted a single-center observational retrospective study based on prospectively collected data…” |
| Setting | 5 | Describe the setting, locations, and relevant dates, including periods of recruitment, exposure, follow-up, and data collection | 4 | Setting: Reproductive Biology Laboratory-CECOS, Rouen University Hospital. Recruitment Sept 1, 2021–Dec 31, 2022; consultations from Jan 24, 2022; data collection through July 31, 2023. |
| Participants | 6 | (*a*) Give the eligibility criteria, and the sources and methods of selection of participants. Describe methods of follow-up | 4, 6 | “Eligible patients included all women who contacted… to register on the waiting list…” Follow-up steps (counselling → ovarian reserve assessment → 2nd consultation → COH → retrieval) quantified in Figure 1. |
|  |  | (*b*) For matched studies, give matching criteria and number of exposed and unexposed | N/A |  |
| Variables | 7 | Clearly define all outcomes, exposures, predictors, potential confounders, and effect modifiers. Give diagnostic criteria, if applicable | 4–6, 19 | Ovarian reserve variables (AMH, FSH, LH, E2, AFC) and COH/vitrification variables defined in Methods; oocyte-stage outcomes defined again in Table 3-5 footnotes. |
| Data sources/ measurement | 8* | For each variable of interest, give sources of data and details of methods of assessment (measurement). Describe comparability of assessment methods if there is more than one group | 4–5 | Standardized counselling questionnaire (Tables 1-2); hormonal assays and pelvic ultrasound for ovarian reserve; COH drug names/doses and monitoring described in detail. |
| Bias | 9 | Describe any efforts to address potential sources of bias | 8, 13 | Baseline comparison between COH-initiators and non-initiators (age, relationship status, education, knowledge); self-report/selection bias discussed in Strengths and limitations. |
| Study size | 10 | Explain how the study size was arrived at | *—* | All women contacted during the recruitment window were included (consecutive sample, no pre-calculated size) |
| Quantitative variables | 11 | Explain how quantitative variables were handled in the analyses. If applicable, describe which groupings were chosen and why | 6, 8–9 | Age dichotomised at 35 years (French regulatory context); AMH dichotomised at 1.1 ng/mL per the Bologna criteria (Ferraretti et al., 2011). |
| Statistical methods | 12 | (*a*) Describe all statistical methods, including those used to control for confounding | 6 | Student's t-test/ANOVA or Kruskal–Wallis for group comparisons; Spearman's rank correlation for associations; GraphPad Prism v8.00. Univariate/exploratory |
|  |  | (*b*) Describe any methods used to examine subgroups and interactions | 6 | Pre-specified subgroups: COH-initiators vs. non-initiators; age <35 vs. ≥35y; AMH <1.1 vs. ≥1.1 ng/mL. |
|  |  | (*c*) Explain how missing data were addressed | 6,13 | percentages use available-case denominators. |
|  |  | (*d*) If applicable, explain how loss to follow-up was addressed | 6, 8, 13, Fig 1 | Attrition described narratively and in Figure 1 |
|  |  | (*e*) Describe any sensitivity analyses | 6, 9–10 | Correlations repeated at patient level with repeated-measures ANOVA (Table 5) to check robustness of per-cycle findings (Table 4). |
| Results | | | | |
| Participants | 13* | (a) Report numbers of individuals at each stage of study-eg numbers potentially eligible, examined for eligibility, confirmed eligible, included in the study, completing follow-up, and analysed | 6–8 | 117 registered → 13 excluded (age) → 77 booked → 69 attended → 51 attended 2nd consult. → 40 initiated COH → 38 completed ≥1 cycle → 62 complete cycles. |
|  |  | (b) Give reasons for non-participation at each stage | 6, 8; Fig. 1 | Reasons given in text for COH-stage exclusions. Earlier-stage reasons in Figure 1, confirm the figure labels each category. |
|  |  | (c) Consider use of a flow diagram | Fig. 1 | Figure 1 (flow chart) provided; caption p.19, figure on separate page/file. |
| Descriptive data | 14* | (a) Give characteristics of study participants (eg demographic, clinical, social) and information on exposures and potential confounders | 6–8; T1-3 | Sociodemographic/reproductive/professional characteristics (Table 1); motivations/knowledge (Table 2); COH/clinical characteristics (Table 3). |
|  |  | (b) Indicate number of participants with missing data for each variable of interest | T1-2 | Denominators (n) given per questionnaire item in Tables 1-2 |
|  |  | (c) Summarise follow-up time (eg, average and total amount) | 6–8 | Mean time registration→consultation (55.1±43.75 d); consultation→2nd visit (80.2±44.2 d); registration→first retrieval (194.1±73.5 d). |
| Outcome data | 15* | Report numbers of outcome events or summary measures over time | 8 | 572 oocytes retrieved, 539 cryopreserved, 474 mature MII; per-woman means (15.1/13.8/12.1); empty punctures 3.2%; complications (1 OHSS, 1 infection). |
| Main results | 16 | (*a*) Give unadjusted estimates and, if applicable, confounder-adjusted estimates and their precision (eg, 95% confidence interval). Make clear which confounders were adjusted for and why they were included | 8–10; T4–5 | Spearman r and P reported for all correlations; t-test/ANOVA P for group comparisons. Univariate/exploratory |
|  |  | (*b*) Report category boundaries when continuous variables were categorized | 6, 8–9 | Age cut-off 35 years; AMH cut-off 1.1 ng/mL (Bologna criteria, cited). |
|  |  | (*c*) If relevant, consider translating estimates of relative risk into absolute risk for a meaningful time period | N/A | Not applicable - no relative-risk measures were computed. |
| Other analyses | 17 | Report other analyses done—eg analyses of subgroups and interactions, and sensitivity analyses | 8–10 | Age- and AMH-based subgroup analyses (first cycle only); per-cycle vs. per-patient repeated-measures comparison (Table 4 vs. 5). |
| Discussion | | | | |
| Key results | 18 | Summarise key results with reference to study objectives | 10, 14 | Discussion opening paragraph and Conclusion restate findings against the stated primary/secondary objectives. |
| Limitations | 19 | Discuss limitations of the study, taking into account sources of potential bias or imprecision. Discuss both direction and magnitude of any potential bias | 13 | “Strengths and limitations” (§4.6): single-center design, sample size, short follow-up, attrition/selection bias, self-report bias. |
| Interpretation | 20 | Give a cautious overall interpretation of results considering objectives, limitations, multiplicity of analyses, results from similar studies, and other relevant evidence | 10–14 | Discussion §4.1–4.5 interpret findings against prior literature (Gambini 2024, Lainas 2020, Donno 2023); Conclusion tempered per reviewer comments. |
| Generalisability | 21 | Discuss the generalisability (external validity) of the study results | 12–13 | Equity-of-access paragraph (socio-economic/informational gradients despite public funding); Limitations note the cohort's highly educated profile. |
| Other information | | | | |
| Funding | 22 | Give the source of funding and the role of the funders for the present study and, if applicable, for the original study on which the present article is based | 14 | “This research did not receive any specific grant from funding agencies in the public, commercial, or not-for-profit sectors.” |

*Give information separately for exposed and unexposed groups.

**Note:** An Explanation and Elaboration article discusses each checklist item and gives methodological background and published examples of transparent reporting. The STROBE checklist is best used in conjunction with this article (freely available on the Web sites of PLoS Medicine at http://www.plosmedicine.org/, Annals of Internal Medicine at http://www.annals.org/, and Epidemiology at http://www.epidem.com/). Information on the STROBE Initiative is available at http://www.strobe-statement.org.
